# Longitudinal observations of sympathetic neural activity and hemodynamics during 6 months recovery from SARS‐CoV‐2 infection

**DOI:** 10.14814/phy2.15423

**Published:** 2022-09-23

**Authors:** Nina L. Stute, Rachel E. Szeghy, Jonathon L. Stickford, Valesha P. Province, Marc A. Augenreich, Stephen M. Ratchford, Abigail S. L. Stickford

**Affiliations:** ^1^ Department of Health and Exercise Science Appalachian State University Boone North Carolina USA

**Keywords:** autonomic function, cold pressor test, COVID‐19, long‐COVID, MSNA, orthostatic

## Abstract

Cross‐sectional data indicate that acute SARS‐CoV‐2 infection increases resting muscle sympathetic nerve activity (MSNA) and alters hemodynamic responses to orthostasis in young adults. However, the longitudinal impact of contracting SARS‐CoV‐2 on autonomic function remains unclear. The aim of this study was to longitudinally track MSNA, sympathetic transduction to blood pressure (BP), and hemodynamics over 6 months following SARS‐CoV‐2 infection. Young adults positive with SARS‐CoV‐2 reported to the laboratory three times over 6 months (V1:41 ± 17, V2:108 ± 21, V3:173 ± 16 days post‐infection). MSNA, systolic (SBP) and diastolic (DBP) blood pressure, and heart rate (HR) were measured at rest, during a cold pressor test (CPT), and at 30° head‐up tilt (HUT). Basal SBP (*p* = 0.019) and DBP (*p* < 0.001) decreased throughout the 6 months, whereas basal MSNA and HR were not different. Basal sympathetic transduction to BP and estimates of baroreflex sensitivity did not change over time. SBP and DBP were lower during CPT (SBP: *p* = 0.016, DBP: *p* = 0.007) and HUT at V3 compared with V1 (SBP: *p* = 0.041, DBP: *p* = 0.017), with largely no changes in MSNA. There was a trend toward a visit‐by‐time interaction for burst incidence (*p* = 0.055) during HUT, wherein at baseline immediately prior to tilting, burst incidence was lower at V3 compared with V1 (*p* = 0.014), but there were no differences between visits in the 30 HUT position. These results support impairments to cardiovascular health, and potentially autonomic function, which may improve over time. However, the improvements in BP over 6 months recovery from mild SARS‐CoV‐2 infection are likely not a direct result of changes in sympathetic activity.

## Graphical abstract

Over the course of 6 months, individuals previously infected with SARS‐CoV‐2 displayed decreases in resting systolic and diastolic blood pressure, but no changes in resting heart rate, MSNA, or sympathetic transduction to blood pressure.

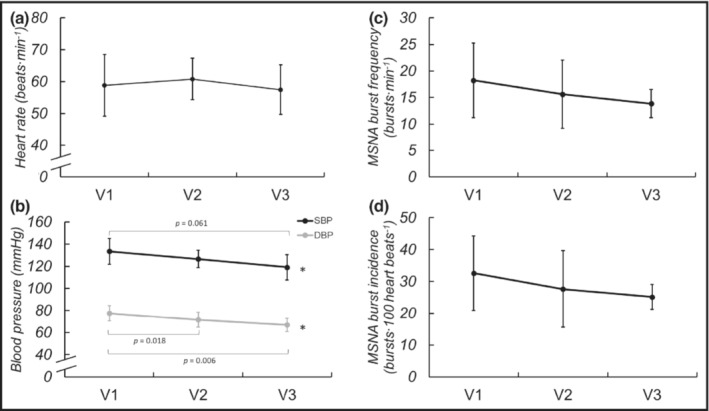

## 1 INTRODUCTION

Infection with the novel severe acute respiratory syndrome coronavirus 2 (SARS‐CoV‐2) results in systemic physiologic decrements, notably within the autonomic nervous system. Cross‐sectional data from our laboratory indicate that acute infection increases resting muscle sympathetic nerve activity (MSNA), dampens the depressor response to sympathetic quiescence, and alters hemodynamic responses to orthostasis in healthy young adults (Stute et al., 2021). However, the time course of recovery and longitudinal impact of contracting SARS‐CoV‐2 on autonomic function remains unclear.

Reports of lingering autonomic and cardiovascular symptoms, sometimes described as components of “long‐COVID,” are becoming more prevalent as the pandemic progresses (Figueroa et al., 2020; Raj et al., 2021). Prolonged clinical sequalae of SARS‐CoV‐2 include symptoms such as elevated resting heart rate and/or heart palpitations, orthostatic intolerance, breathlessness, cognitive impairment, anosmia, generalized pain and discomfort, and fatigue (Eshak et al., 2020; Miglis et al., 2020; Oates et al., 2020; Xiong et al., 2021). Additionally, a number of case studies have described the development of a disorder of the autonomic nervous system—postural orthostatic intolerance (POTS)—following SARS‐CoV‐2 infection (Blitshteyn & Whitelaw, 2021; Raj et al., 2021). The persistence of these autonomic and cardiovascular symptoms may be due to organ injury, chronic inflammation/immune response, deconditioning, psychosocial responses, and/or continued presence of the virus (Raveendran et al., 2021).

The incidence of long‐COVID in non‐hospitalized people infected with SARS‐CoV‐2 is unclear, with reports ranging from ~5 to >50% (Augustin et al., 2021; Cabrera Martimbianco et al., 2021; Dani et al., 2021; Moreno‐Perez et al., 2021). While it is evident, based upon symptomology, that autonomic dysfunction may persist following SARS‐CoV‐2 infection, no investigations have yet reported direct measures of MSNA and sympathetic transduction to blood pressure throughout prolonged recovery from COVID‐19. Thus, the purpose of this investigation was to longitudinally track MSNA activity, reactivity, and transduction to blood pressure (BP), as well as hemodynamic parameters, over 6 months of recovery from SARS‐CoV‐2 in otherwise healthy young adults. We hypothesized that MSNA at rest and during passive head‐up tilt would decline throughout recovery from the viral infection. We also hypothesized an increase in the depressor responses to non‐bursting cardiac cycles over the course of recovery.

## 2 METHODS

### 2.1 Ethics statement

All participants were informed of the study purpose and protocols, and they gave written informed consent to protocols approved by the Institutional Review Board of Appalachian State University (#20‐0304). The study was performed in accordance with the ethical standards described by the Declaration of Helsinki.

### 2.2 Study design

Otherwise healthy young adults who tested positive for SARS‐CoV‐2 using a nasopharyngeal swab polymerase chain reaction (PCR) assay reported to the laboratory three times (V1, V2, V3), beginning approximately 1 month following their positive test, over the course of 6 months. All participants were free from cardiovascular, metabolic, or renal disease, and female participants were not currently pregnant or breastfeeding. Participants did not require hospitalization during or following infection.

For testing, participants arrived at the laboratory in a fasted state, having abstained from exercise, caffeine, and alcohol for at least 24 h before testing, and ≥4 h after a snack or light meal. Testing took place in a quiet, environmentally controlled laboratory, with an ambient temperature of ~23°C.

*COVID‐19 Symptom Severity Survey*. On the day of testing, subjects ranked their COVID‐19 symptoms on a scale of 0–100 with increasing severity, which can be found elsewhere (Stute et al., 2021). Measured symptoms included: chest pain, chills, diarrhea, dizziness or vertigo, dry cough, dry eyes, dry mouth, fatigue, fever over 37.9°C, headache, lack of appetite, loss of smell or taste (anosmia), muscle or body aches, nasal congestion or runny nose, nausea or vomiting, shortness of breath, difficulty breathing, dyspnea, sore joints, or sore throat. The values for all symptoms were totaled and averaged for an average symptom severity score. Mild severity scores were categorized as a score of 0–33, moderate from 34–66, and severe from 67–100.

### 2.3 Experimental measures

Participants were in the supine position on a bed for instrumentation. Multiunit MSNA was assessed using the microneurographic technique, as previously described (Vallbo et al., 1979; Wallin et al., 1974; White et al., 2015). Briefly, a recording electrode was inserted in the peroneal nerve at the fibular head, and a reference electrode was inserted subcutaneously 2–3 cm from the recording electrode. The nerve signals were amplified (70,000–160,000‐fold), band‐pass filtered (700–2000 Hz), full‐wave rectified, and integrated with a resistance‐capacitance circuit (time constant 0.1 s). Criteria for adequate MSNA recording included: (a) pulse synchrony; (b) increases in response to breath‐holding; and (c) insensitivity to gentle skin touch or a loud noise.

Heart rate (HR) determined from lead II of the electrocardiogram (Biopac Systems), beat‐by‐beat BP measured by finger photoplethysmography (NOVA; Finapres Medical Systems), and MSNA (662C‐4; Department of Biomedical Engineering, University of Iowa) were continuously recorded during all tests. Arm cuff systolic (SBP) and diastolic (DBP) pressures were measured by electrosphygmomanometry (NOVA; Finapres Medical Systems) at specified time points during each test.

After an acceptable nerve recording site had been found and following >10 min of supine rest, basal data were collected during spontaneous breathing for 5 min.

The participant then performed a cold pressor test (CPT). For the CPT, baseline data of 1 min were collected and the participant's hand was then immersed in an ice water bath for 2 min. Participants were instructed to avoid breath holding during the test. After the test, the participant's hand was immediately dried and wrapped in warmed towels during a 3‐min period of recovery. The CPT was used to assess the central integration of vasomotor sympathetic processes and their efferent pathways (Seals, 1990; Victor et al., 1987). Immediately following recovery from the CPT, participants were asked to rate their perception of pain on a numeric rating scale of 1 to 10, with 1 being no pain and 10 being the worst pain imaginable (Bijur et al., 2003; Serlin et al., 1995).

Following the CPT, when the participant's HR and BP were within 5 bpm and 5 mmHg from baseline values (~5 min, depending on subject) they were subsequently tilted passively to 30° and 60° head‐up tilt (HUT) for 5 min each. A belt was placed across the participants’ waist to ensure that they would not fall. A bicycle saddle was used to support approximately two‐thirds of the participant's body weight during tilt, allowing their leg to be relaxed for microneurography.

### 2.4 Data analysis

Sympathetic and hemodynamic data were sampled at 625 Hz with a commercial data acquisition system (Biopac Systems). MSNA bursts were identified using computer software (LabView Software; National Instruments) with a 3:1 signal‐to‐noise ratio threshold within a 0.5 s search window and an expected burst reflex latency of 1.3 s from the preceding R‐wave (Cui et al., 2001). All bursts were confirmed by an experienced microneurographer, and re‐running of the data with sound (to listen for sympathetic bursts from the raw signal) was performed using Spike2 8.08 software to confirm any questionable bursts. Within the integrated neurogram, the burst with the largest amplitude during baseline was assigned a value of 100, and all bursts in that trial (e.g., spontaneous breathing, baseline prior to CPT) were normalized to that burst (Joyner & Halliwill, 2000). In this same manner, calibration bursts were assigned during the baseline periods preceding each test (i.e., CPT, HUT). Burst areas of the integrated neurogram, systolic and diastolic pressures, and R‐R interval were measured simultaneously on a beat‐to‐beat basis. Total activity of the burst was defined as the burst area of the rectified and integrated neurogram. The number of bursts per minute (burst frequency) and per 100 heartbeats (burst incidence) and total burst area per minute (total activity) were used as quantitative indices of MSNA.

During spontaneous breathing, continuously recorded variables (i.e., MSNA, HR, beat‐by‐beat BP) were averaged over the entire 5‐min period. Arm cuff BP was measured at minutes 1 and 4 of spontaneous breathing, and averaged. Estimated cardiac output was calculated using the Windkessel formula (Koenig et al., 2015).

During the CPT, continuously recorded variables were averaged over the 1‐min baseline period, each 30 s period during CPT, and each minute during recovery from CPT. Arm cuff BP was measured once during baseline prior to CPT, each minute during the CPT, and at 90 s into the subsequent recovery.

During HUT, continuously recorded variables were averaged over the 1‐min baseline period and over the entire 5‐min tilting period. All variables were recorded during 30° HUT, but only HR data were available during 60° HUT due to technical difficulties. Arm cuff BP was measured once during baseline prior to HUT, and at minutes 1 and 4 of 30° HUT.

Sympathetic transduction, which was assessed during spontaneous breathing (i.e., basal data collection) was calculated using an open‐source program by O'Brien et al. (2021). Briefly, electrocardiogram, arterial pressure, and MSNA signals (i.e., burst/no burst) from the 5‐min resting period were time aligned. The absolute (mmHg) and relative (%) changes (Δ) in mean arterial pressure MAP (MAP) were then determined for each of 12 consecutive cardiac cycles following an MSNA burst (change calculated from the cardiac cycle in which the burst occurred). The average ΔMAP was determined for each cardiac cycle, and the largest value was used as the measure of sympathetic transduction. Similarly, pressor responses following “non‐bursts” (i.e., cardiac cycles absent of MSNA bursts, or sympathetic quiescence) were determined by tracking ΔMAP for 12 cardiac cycles following a non‐bursting cardiac cycle. The average ΔMAP was determined for each cardiac cycle, and the nadir change was used as the measure of the pressor response to sympathetic quiescence.

Estimates of cardiovagal baroreflex sensitivity (cBRS) and sympathetic baroreflex sensitivity (sBRS) were assessed during spontaneous breathing. cBRS was determined using the slope of the linear correlation between R‐R interval and beat‐by‐beat SBP in each participant. sBRS was assessed using the slope of the linear correlation between MSNA burst incidence and beat‐by‐beat DBP in each participant as described previously (Okada et al., 2012), using 3‐mmHg DBP bin increments.

### 2.5 Statistical analysis

Statistical analysis was performed using commercially available software (IBM SPSS Statistics Version 26; IBM Corp.; SAS Version 9.4; SAS Institute). Continuous variables were checked for normality using Kolmogorov–Smirnov tests, and log transformations were made for data not conforming to normal distribution. Repeated measures analysis of variance (ANOVA) was used to assess the effect of visit on hemodynamic resting outcome variables. Linear mixed models were used to assess the effect of visit on resting MSNA outcome variables. Two‐way repeated‐measures ANOVA was used to assess the effect of visit on CPT (visit × time) and HUT (visit × position) hemodynamic responses. Linear mixed models were used to assess the effect of visit and time (CPT) or position (HUT) MSNA responses. Bonferroni post hoc analysis was conducted when interactions were identified. Levene's test was used to assess the equality of variances. Pearson correlations were used to determine if relationships existed between changes in sympathetic and BP measures over time. Statistical significance was set at *p <* 0.05. Data are expressed as means ± standard deviation (SD).

## 3 RESULTS

Ten participants (3F/7M, 20.5 ± 1.2 year, 73 ± 11 kg, 175 ± 10 cm) completed this longitudinal study. The three visits took place at 41 ± 17, 108 ± 21, and 173 ± 16 days post positive PCR test. Four participants received SARS‐CoV‐2 vaccinations during the time course of the study, three of whom received both doses: Pfizer (*n* = 1, 38 [dose 1] and 20 days [dose 2] prior to V2), and Moderna (*n* = 2, 13 days prior to V1 [dose 1], 20 days prior to V3 [dose 2] and 39 [dose 1] and 13 days [dose 2] prior to V2). One participant received one dose of Pfizer during the study (16 days prior to V3). Six participants did not receive vaccines during the study. Study visits were buffered by >10 days following vaccination to minimize any residual inflammation.

### 3.1 SARS‐CoV‐2 Symptom Severity Survey

Average symptom severity score and number of participants with symptoms are presented in Table 1. One female was asymptomatic and was not included in the symptom severity survey.

**TABLE 1:** SARS‐CoV‐2 symptom severity survey

Symptom	Visit 1 (43 ± 16 days) (7 M/2F)	Visit 2 (103 ± 14 days) (7 M/2F)	Visit 3 (168 ± 16 days) (7 M/2F)
Chest pain	1 ± 4 (1F)	0	0
Chills	3 ± 7 (1 M)	0	0
Diarrhea	0	0	0
Dizziness/Vertigo	0	0	0
Dry cough	1 ± 4 (1 M)	0	0
Dry eyes	3 ± 7 (1 M)	3 ± 7 (1 M)	0
Dry mouth	2 ± 5 (1 M)	3 ± 5 (2 M)	0
Fatigue	6 ± 8 (2 M/1F)	0	0 ± 1 (1F)
Fever over 100.3 °F	0	0	0
Headache	0	0	0 (3F)
Lack of appetite	1 ± 4 (1 M)	0	0 ± 1 (1F)
Loss of smell/taste, anosmia	6 ± 12 (1 M/1F)	0 ± 1 (1F)	0 ± 1 (1F)
Muscle or body aches	2 ± 4 (2 M)	0	0
Congestion or runny nose	2 ± 4 (2 M)	1 ± 4 (1 M)	0 (1 M)
Nausea or vomiting	0	0	0
Shortness of breath, difficulty breathing, dyspnea	0 (4F)	0	0
Sore joints	3 ± 7 (1 M)	0	0
Sore throat	3 ± 5 (2 M)	0	0
Number of symptoms	3 ± 2	0.7 ± 0.8	0.6 ± 1
Average Severity Score	1.72 ± 5.08	0.38 ± 2.25	0.05 ± 0.31

*Note*: Data are means ± SD. Header depicts total sample sizes available during each visit, while sample sizes within specific variables depicts how many participants had individual symptoms. One female participant was asymptomatic and excluded from the table.

### 3.2 Basal sympathetic activity and hemodynamics

**FIGURE 1:**
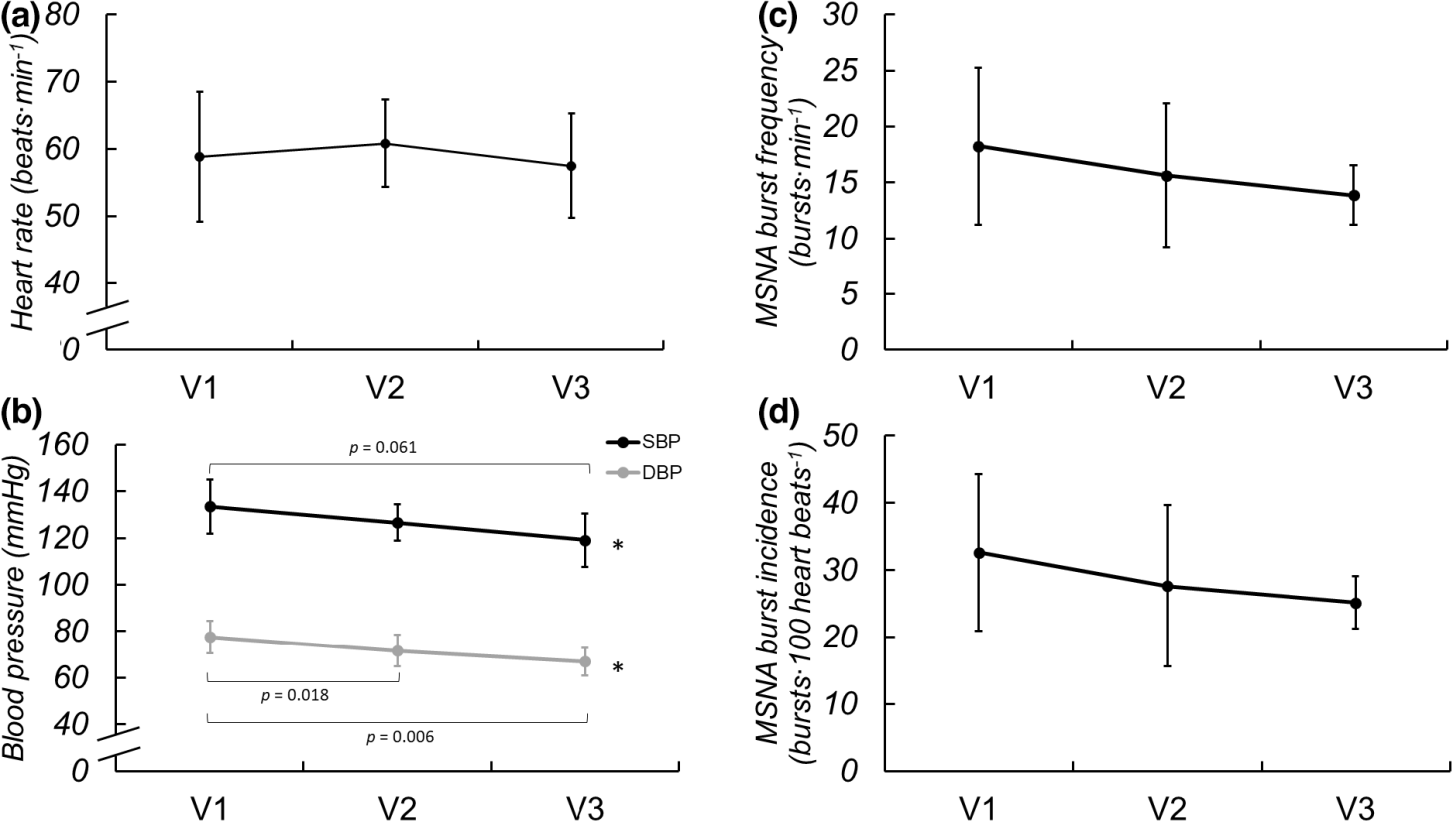
Basal HR (a), SBP and DBP (b), MSNA burst frequency (c) and burst incidence (d) during spontaneous breathing at V1 (41 ± 17 days post‐positive test, *n* = 10/8 for hemodynamic and MSNA data, respectively), V2 (108 ± 21 days post‐positive test, *n* = 10/8), and V3 (173 ± 16 days post‐positive test, *n* = 10/7) following SARS‐CoV‐2 infection. Repeated measures ANOVA indicated no significant change in HR over time (*p* = 0.151) but SBP (*p* = 0.019) and DBP (*p* < 0.001) significantly decreased. Mixed model analyses indicated no significant changes in burst frequency (*p* = 0.143) or incidence (*P* = 0.199) over the ~6 months recovery. *P < 0.05 for visit. Reported pairwise comparisons are adjusted for multiple comparisons (*Bonferroni*). Data are mean ± SD. MSNA, muscle sympathetic nerve activity.

MSNA was longitudinally obtained on eight of the participants (1F/7M). Basal MSNA, HR, and BP were collected during 5 min of spontaneous breathing and are displayed in Figure 1. Basal MSNA burst frequency (*p* = 0.143), incidence (0.199), and total activity (*p* = 0.474) did not significantly change across visits. Basal HR also was not different across visits (*p* = 0.151), but SBP (*p* = 0.019) and DBP (*p* < 0.001) decreased throughout recovery. Estimates of cBRS did not significantly change (V1: 0.418 ± 6.54, V2: 1.362 ± 83.749, V3: 3.085 ± 5.336, *p* = 0.584). There were no significant differences in estimated cardiac output between visits (V1: 6.5 ± 1.2, V2: 6.7 ± 1.0, V3: 5.9 ± 1.0 L/min*, p* = 0.131). The change in MSNA from V1 to V3 was not correlated with the change in SBP (*r* = −0.009, *p* = 0.984) or DBP (*r* = 0.113, *p* = 0.810). Further, neither absolute (*p* = 0.252) nor relative (*p* = 0.435) sympathetic transduction to blood pressure changed across visits. Similarly, the absolute (*p* = 0.169) and relative (*p* = 0.127) changes in BP in response to non‐bursting cardiac cycles were similar across time. Estimates of sBRS also did not change across visits (V1: −0.918 ± 0.890, V2: −0.597 ± 1.411, V3: −0.846 ± 0.917, *p* = 0.832).

### 3.3 Responses to cold pressor test

**FIGURE 2:**
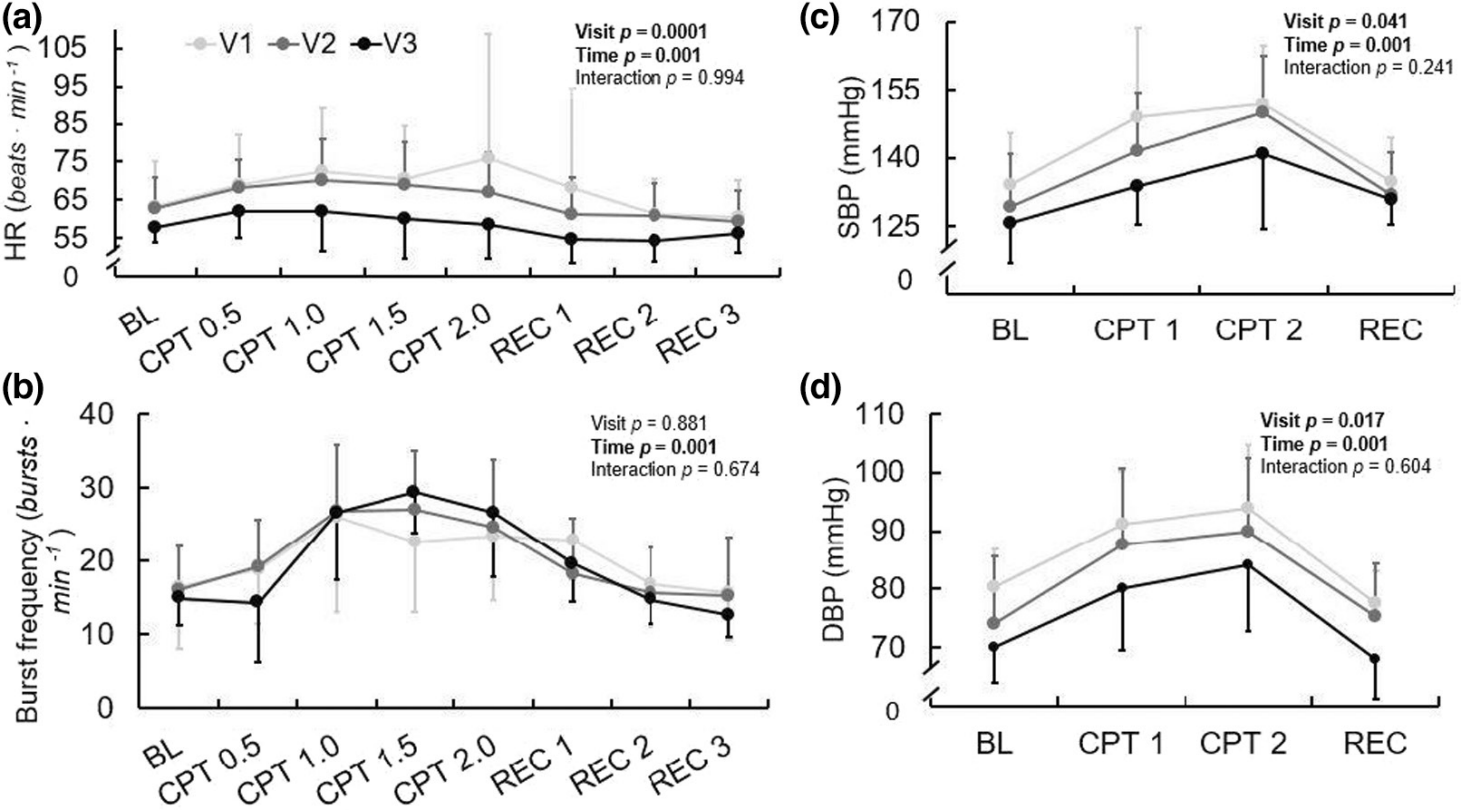
HR (a), MSNA burst incidence (b), SBP (c), and DBP (d) response to CPT—including 1‐min baseline, every 30 s during CPT, every 1 min during recovery—at V1 (41 ± 17 days post‐positive test, *n* = 10/6 for hemodynamic and MSNA data, respectively), V2 (108 ± 21 days post‐positive test, *n* = 10/6), and V3 (173 ± 16 days post‐positive test, *n* = 10/6) during recovery from SARS‐CoV‐2 infection. Repeated measures ANOVA indicated no significant change in HR response over time, but there was a significant effect of time on SBP and DBP during CPT. Mixed model analyses indicated no significant changes in MSNA burst frequency (*p* = 0.881) over the ~6 months recovery. *p < 0.05 for visit. Reported pairwise comparisons are adjusted for multiple comparisons (*Bonferroni*). Data are mean ± SD. MSNA, muscle sympathetic nerve activity. *p < 0.05 for visit. †*p* < 0.05 compared with V1 at position. BL, baseline; CPT, cold pressor test; DBP, diastolic blood pressure; MSNA, muscle sympathetic nerve activity; REC, recovery; SBP, systolic blood pressure.

Results of the CPT are shown in Figure 2. There was no effect of visit (*p* = 0.188) or visit‐by‐time interaction (*p* = 0.085) on the HR response to CPT. However, there were significant main effects of visit on the SBP (*p* = 0.041) and DBP (*p* = 0.017) responses to CPT, with DBP being lower at V3 compared with V1.

There were no effects of visit or visit‐by‐time interactions on MSNA burst frequency (*p* = 0.889, *p* = 0.673) or burst incidence (*p* = 0.424, *p* = 0.237). While there was not a visit‐by‐time interaction on total MSNA (*p* = 0.993), there was a significant effect of visit (*p* = 0.0002). Namely, the total MSNA differed between both V1 and V2 (*p* = 0.002) with total MSNA decreasing, and V2 and V3 (*p* = 0.006), with total MSNA increasing. Ratings of pain during the CPT tended to increase but did not significantly change across visits (V1: 4.8 ± 2.0 a.u., V2: 5.4 ± 1.8 a.u., V3: 5.8 ± 2.5 a.u., *p* = 0.072).

### 3.4 Responses to orthostatic challenge

**FIGURE 3:**
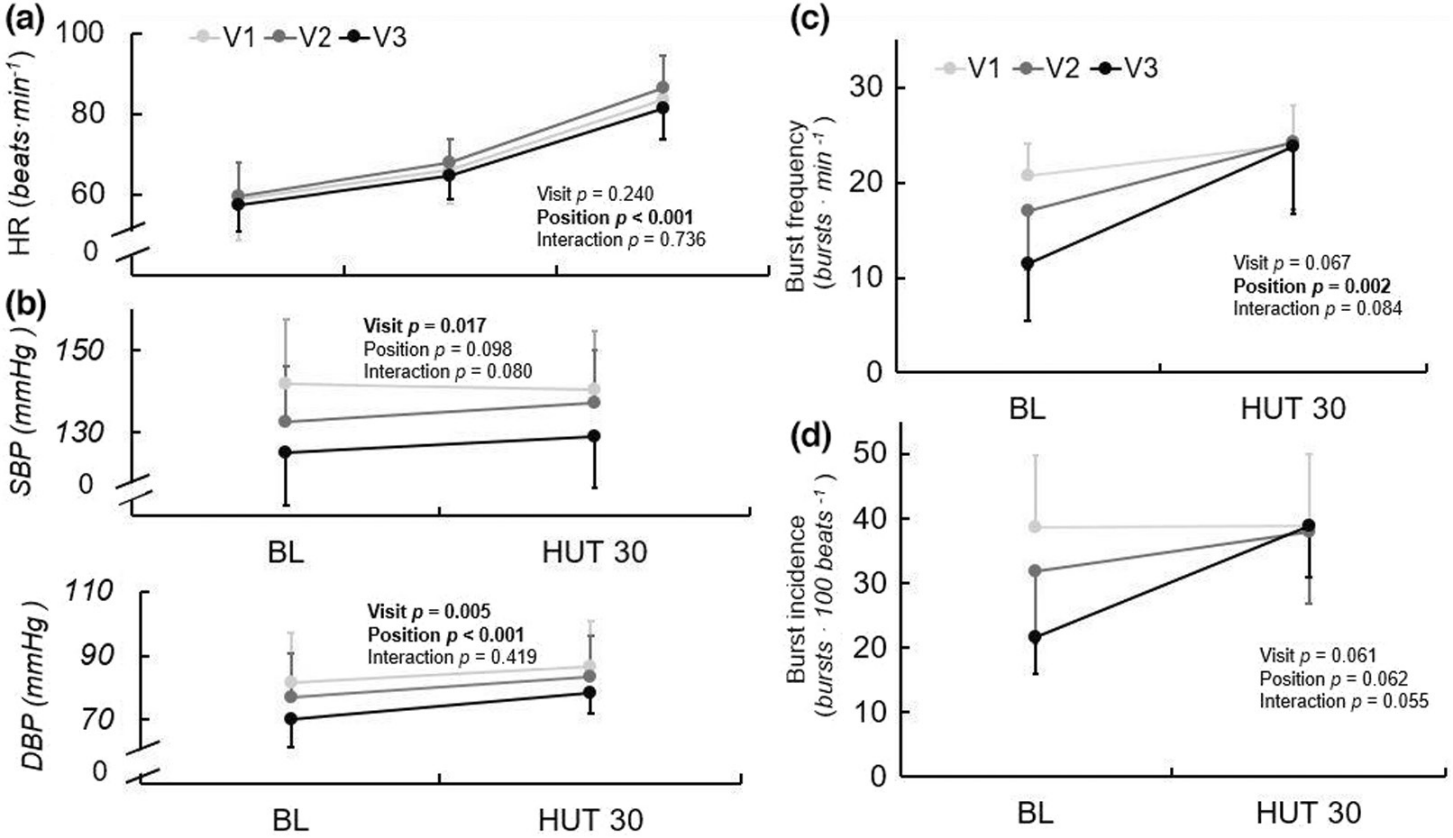
HR (a), SBP and DBP (b), MSNA burst frequency (c) and burst incidence (d) response to 30° and 60° (*HR only*) HUT at V1 (41 ± 17 days post‐positive test, *n* = 10/6 for hemodynamic and MSNA data, respectively), V2 (108 ± 21 days post‐positive test, *n* = 10/6), and V3 (173 ± 16 days post‐positive test, *n* = 10/6) during recovery from SARS‐CoV‐2 infection. Repeated measures ANOVA indicated no significant change in HR response over time, but SBP and DBP during the HUT protocol significantly decreased. Mixed model analyses indicated a significant effect of position for MSNA burst frequency (*p* = 0.002), and there were also trends toward a significant effect of visit on burst frequency (*p* = 0.067) and visit‐by‐position interaction for burst incidence (*p* = 0.055) over the ~6 months recovery. Data are mean ± SD. *p < 0.05 for visit. †*p* < 0.05 compared with V1 at position. BL, baseline; DBP, diastolic blood pressure; HUT, head up tilt; MSNA, muscle sympathetic nerve activity; SBP, systolic blood pressure.

Responses to 30° head‐up tilt are displayed in Figure 3. Response to 60° HUT is displayed for HR only. The HR response to HUT did not change across visits. There was a significant effect of visit on SBP during HUT (*p* = 0.017), with post hoc testing indicating decreased SBP during V3 compared with V1 (*p* = 0.016). Similarly, there was a significant effect of visit on DBP during HUT (*p* = 0.005), wherein DBP was lower during V3 compared with V1 (*p* = 0.007).

There were no significant effects of visit or visit‐by‐time interactions for MSNA burst frequency (*p* = 0.067, *p* = 0.084), burst incidence (*p* = 0.061, *p* = 0.055), or total MSNA (*p* = 0.720, *p* = 0.216) during HUT. There was a trend toward a visit‐by‐time interaction for burst incidence (*p* = 0.055) during HUT, wherein at baseline immediately prior to tilting, burst incidence tended to be lower at V3 compared with V1(*p* = 0.014), but there were no differences between visits in the 30 HUT position. At V1 there was an increase of 3.3 bursts∙min^−1^ from supine to 30° HUT, whereas there were increases of 7.3 and 12.3 bursts∙min^−1^ at V2 and V3, respectively. At V1 there was an increase of 0.15 bursts∙100 heartbeats^−1^ from supine to 30° HUT, whereas there were increases of 6.2 bursts∙100 heartbeats^−1^ and 17.2 bursts∙100 heartbeats^−1^ at V2 and V3, respectively.

## 4 DISCUSSION

The current longitudinal study was designed to elucidate the potential changes in sympathetic neural and hemodynamic parameters over 6 months of recovery from SARS‐CoV‐2 infection. The primary findings are that MSNA during a supine resting protocol does not significantly change throughout recovery from SARS‐CoV‐2 infection, though some baseline measures of sympathetic activation prior to sympathoexcitatory challenges (i.e., CPT, HUT) indicate that resting MSNA may, indeed, be decreasing over the course of recovery. Further, BP was consistently reduced at rest, as well as during CPT and HUT, from early (41 ± 17 days) compared with later (173 ± 16 days) in recovery. Our hypotheses, however, were largely incorrect, as we did not observe a reduction in MSNA reactivity to stressors nor did we find a greater depressor response to sympathetic quiescence over the course of recovery. These results suggest that improvements in BP at rest and during physiological stress over 6 months recovery from mild SARS‐CoV‐2 infection may not be a result of changes in sympathetic neural activation.

### 4.1 Resting sympathetic activity and hemodynamics

Our prior work investigating the acute sympathetic neural impact of SARS‐CoV‐2 in this same population showed augmented resting MSNA in young adults recently diagnosed with SARS‐CoV‐2 when compared with healthy control participants (Stute et al., 2021). In the present study we did not see a decrease in resting MSNA, but BP—perhaps of greater clinical functionality with regard to cardiovascular health—did decrease throughout recovery (Figure 1). The lack of significant changes observed in MSNA during spontaneous breathing over 6 months following infection could be due to a variety of factors, including prolonged elevation in inflammatory cytokines that accompany SARS‐CoV‐2 infection. As seen in both animal and human models, inflammatory cytokines are elevated during SARS‐CoV‐2 infection (e.g., C‐reactive protein, tumor necrosis factor alpha, interlukin‐6, etc.) and are hypothesized to elicit marked increases in MSNA, evidenced by chronic inflammatory conditions like obesity, rheumatoid arthritis, and others (Adlan et al., 2017; Niijima et al., 1991; Zhang et al., 2003).

Further, because resting MSNA has inherently high inter‐individual variability (Keir et al., 2020), detecting statistically significant changes can be difficult. However, resting MSNA tends to be relatively consistent within an individual (Fagius & Wallin, 1993; Hissen et al., 2015; Sundlof & Wallin, 1977); thus, our longitudinal design strengthens our ability to interpret the impact of SARS‐CoV‐2 infection on sympathetic activity. Five of our eight participants who completed longitudinal MSNA testing had their highest burst frequency at V1, with an average individual decrease over time of 6.3 bursts∙min^−1^, whereas the three participants whose highest MSNA was *not* at V1 had an average individual increase of only 2.0 bursts∙min^−1^, which could simply be attributed to day‐to‐day variability.

However, despite the lack of significant changes in resting MSNA over 6 months, SBP and DBP were consistently decreasing among our participants over the course of recovery. These seemingly divergent findings could be explained by the age of our cohort, as young adults generally do not exhibit a relationship between MSNA and arterial pressure (Matsukawa et al., 1998; Narkiewicz et al., 2005). Indeed, in the current study, there were no significant correlations between MSNA and BP at any time point, nor in the changes in MSNA and BP from V1 to V3. Notably, there is a positive relationship between MSNA and BP in older populations (Hart et al., 2012). Thus, we may have expected to observe concomitant reductions in sympathetic activity and BP if the current study had included older adults recovering from SARS‐CoV‐2. The reductions in resting BP in the present study could be due to improvements in endothelial function (Ratchford et al., 2021) and/or decreased arterial stiffness (Szeghy et al., 2021), as assessed by carotid‐femoral pulse wave velocity (Szeghy et al., 2022) over 6 months recovery in this cohort. No changes in estimated cardiac output were observed over time in this cohort, further supporting the potential role of changes in endothelial function and/or arterial stiffness in observed reductions in BP in the months following SARS‐CoV‐2 infection. Interestingly, even though BP decreased while MSNA remained largely unchanged (which could suggest changes in adrenergic receptor sensitivity), measures of sympathetic transduction to BP and sympathetic baroreflex sensitivity also did not change during recovery from SARS‐CoV‐2 infection. Taken together, these results indicate that improvements in BP at rest over 6 months recovery from mild SARS‐CoV‐2 infection likely are not a result of changes in sympathetic neural activation or transduction.

### 4.2 Responses to cold pressor test

The CPT is commonly used to elicit increases in arterial BP and MSNA, which allows evaluation of non‐baroreflex‐mediated sympathetic neural control (Lamotte et al., 2021; Victor et al., 1987; Yamamoto et al., 1992). During a CPT, healthy individuals typically exhibit increases in MSNA and subsequent augmentation of BP, and the current cohort displayed normal reactive MSNA and BP changes to the CPT throughout 6 months following SARS‐CoV‐2 infection. However, both SBP and DBP during the CPT were significantly decreasing throughout the 6 months following infection. Interestingly, using a linear mixed model statistical approach, we found that total MSNA decreased from V1 to V2, but divergently increased from V2 to V3, without concomitant changes in pressures. These findings are likely a combined result of (a) one participant who exhibited a very large total MSNA response to CPT during V3, (b) a small sample size, especially at V3, and (c) the linear mixed model approach, which accounted for attrition but consequently included different participants at each time point. Further, while total MSNA values are relative to each participant and normalized, there is inherent variability in total MSNA with a repeated measures design; for example, the microelectrode placement will vary from visit to visit, as will the quality of the neurogram itself. These limitations in the experimental measure may have impacted the current finding of increased total MSNA during CPT from V2 to V3.

Notably, while not significantly increased, we observed a tendency toward increasing ratings of CPT pain throughout recovery. In our initial investigation, individuals recovering from SARS‐CoV‐2 displayed markedly lower ratings of pain during the CPT compared with control participants. Pain is a hallmark symptom reported in individuals with acute SARS‐CoV‐2 infection, as well as in long‐COVID (Augustin et al., 2021; Carfi et al., 2020). However, a growing number of case studies have reported reductions in chronic disease‐related pain during and following infection with SARS‐CoV‐2 (Hentsch et al., 2021). Further, some evidence exists to suggest an analgesic role for the SARS‐CoV‐2 spike protein via blocking of vascular endothelial growth factor‐A signaling (Moutal et al., 2021). Pain perception and MSNA responses during a painful stimulus have been shown to be positively related (Huang et al., 2021). Though, Watso et al. found that administration of ketamine, a potent analgesic, in healthy individuals decreased ratings of CPT pain without any significant effect on MSNA (Watso et al., 2021). Thus, it is possible that changes in pain but not MSNA could be observed during a CPT over an extended investigation period. However, neither variable significantly changed in the current study of healthy participants.

### 4.3 Responses to orthostatic challenge

We previously observed higher MSNA throughout an orthostatic challenge (30 and 60° HUT), as well as an exaggerated HR response to orthostasis, in young adults during acute (~1 month) recovery from SARS‐CoV‐2 compared with healthy control participants (Stute et al., 2021). However, the longitudinal data from the current study indicate largely similar measures of MSNA in the 30° HUT position and no differences in the HR response throughout 6 months of recovery. The discrepancy in MSNA responses may arise from differences in the degree of orthostatic challenge (30 and 60° HUT in the acute study vs. only 30° HUT in the current). Nevertheless, this does not explain the lack of change in HR response, as HR data were examined for both 30 and 60° HUT at every visit. This finding suggests that either (a) the HR response to orthostasis remains exaggerated compared with normal for at least 6 months following infection, or (b) the greater HR response during HUT that we initially observed in SARS‐CoV‐2 compared with healthy control participants was driven by factors other than the viral infection.

Lockdowns characteristic of the COVID‐19 pandemic have been shown to adversely influence heart rate variability, wherein resting HR and indices of sympathetic input are elevated following lockdown (Bourdillon et al., 2020). The added psycho‐social stressors of lockdown and quarantine measures could certainly impact overall mental and physical health (Amerio et al., 2021; Zaccagni et al., 2021), which may have subsequently affected cardiovascular responses in this cohort.

The trend toward significance in burst incidence response during HUT was driven by decreased levels of *baseline* MSNA prior to tilting at V3 compared with V1. Thus, while basal MSNA during supine spontaneous breathing did not significantly change across visits, we again observed potential evidence of decreasing baseline MSNA prior to a stressor from early to late recovery. Interestingly, at the 30° HUT position, average values of MSNA burst frequency (24 vs. 24 vs. 24 bursts∙min^−1^) and incidence (39 vs. 38 vs. 39 bursts∙100 heart beats) were nearly identical across visits, suggesting perhaps a maximal upper limit or, alternatively, a minimum required level of sympathetic activation to maintain BP during mild orthostasis. Orthostasis causes a shift in central blood volume, which, typically, results in a baroreceptor‐mediated increase in MSNA (Ichinose et al., 2006; Rowell, 1993). However, at V1, there were minimal changes in MSNA indices from HUT baseline to 30° HUT. Certainly, the 30° HUT position does not produce as great of a shift in blood volume as a more upright position, but this is still a considerable fluid shift; indeed, Kamiya et al. (2009) found that far more conservative increases in the upright position compared with the current protocol resulted in increases in MSNA. Thus, an increase in MSNA is expected during 30° HUT compared with the supine position. The results of the current study, wherein MSNA was largely unchanged in response to orthostasis at V1, suggest a potential increase in baroreflex sensitivity throughout recovery from SARS‐CoV‐2 infection.

### 4.4 Limitations

The current study had several limitations that should be addressed. First, there have been noted increases in levels of anxiety and depression reported among college‐aged individuals during the COVID‐19 pandemic (Charles et al., 2021; Copeland et al., 2021), which can negatively impact cardiovascular health (Cohen et al., 2015; Holwerda et al., 2018). As we did not assess any indices of mental health, interpretation of the data is limited by the potential role of mental health changes on the cardiovascular variables assessed. However, it should be noted that medication usage did not change throughout the study, suggesting no major alterations to mental health that warrant diagnosis and prescription drug therapies. For sympathetic transduction to BP, we did not measure vascular conduction via Doppler ultrasound (i.e., the gold standard for neurovascular transduction) and instead used the more “indirect” calculation of MSNA to BP relationship—perhaps limiting our interpretation of this measure. Additionally, we did not obtain baseline data on participants prior to SARS‐CoV‐2 infection or SARS‐CoV‐2‐negative time control group, which limit our interpretation of the observed changes.

However, our findings are strengthened by the study's longitudinal repeated‐measures design. The attrition associated with this longitudinal design, though, in addition to challenges in obtaining microneurographic data on each participant at each time point, limited our sample size and statistical power. Lastly, we had an unequal sex distribution of our participants (3F/7M), as well as within our MSNA subset (1F/7M). This certainly limited our ability to investigate possible sex differences in the sympathetic neural recovery from SARS‐CoV‐2, and our findings may not be generalizable to young females recovering from this viral infection.

## 5 CONCLUSION

Neurological manifestations of SARS‐CoV‐2 infection have been widely reported. Indeed, we previously observed elevated sympathetic neural activation acutely (~1 month) following the viral infection compared with a group of control participants. The current study is the first to provide longitudinal measures of both autonomic and hemodynamic function at rest and in response to physiological stress following SARS‐CoV‐2 infection. Our findings indicate that resting and reactive BP, but not HR, decrease over the course of recovery. Further, while some indices of resting sympathetic activity appear to decrease over time, there were largely no significant changes in basal MSNA during supine spontaneous breathing throughout 6 months following initial SARS‐CoV‐2 infection. These results suggest that improvements in BP at rest and during physiological stress over 6 months recovery from mild SARS‐CoV‐2 infection are likely not a result of changes in sympathetic neural activation. Notably, these responses were observed in young, healthy participants with mild cases of COVID‐19, and the course of autonomic function recovery in more severe disease and/or more vulnerable populations is unknown.

## AUTHOR CONTRIBUTIONS

J.L.S, S.M.R., A.S.L.S. conceived and designed research; N.L.S., R.E.S. V.M.P., M.A.A., J.L.S., S.M.R., A.S.L.S. performed experiments; N.L.S., R.E.S., J.L.S., S.M.R., A.S.L.S. analyzed data; N.L.S., R.E.S., V.M.P., M.A.A., J.L.S., S.M.R., A.S.L.S. interpreted results of experiments; N.L.S., A.S.L.S prepared figures; N.L.S., A.S.L.S. drafted manuscript; N.L.S., R.E.S., V.M.P., M.A.A., J.L.S., S.M.R., A.S.L.S. edited and revised manuscript; N.L.S., R.E.S., V.M.P., M.A.A., J.L.S., S.M.R., A.S.L.S. approved final version of manuscript.

## FUNDING INFORMATION

This study was partially supported by an internal COVID‐19 Research Cluster Award at Appalachian State University.

## CONFLICT OF INTEREST

The authors have no conflicts of interest to declare.

## ETHICS STATEMENT

The study protocol in this study complied with the Declaration of Helsinki and the principles of Good Clinical Practice, and was approved by the Ethics Committee of Appalachian State University (#20‐0304).
